# New Analytical Approach for the Determination of Calcium Phosphate Dibasic and Tribasic in Processed Food by Comparison of Ion Chromatography with High-Performance Liquid Chromatography

**DOI:** 10.3390/foods9030248

**Published:** 2020-02-25

**Authors:** Minjung Song, Juhee Park, Jihyun Lee, Heejae Suh, Hyunjung Lee, Dojin Ryu, Chan Lee

**Affiliations:** 1 Advanced Food Safety Research Group, BrainKorea21 Plus, Department of Food Science and Technology, Chung-Ang University, 4726, Seodong-daero, Anseong-si 17546, Korea; gkfka27@cau.ac.kr (M.S.); jh0204@cau.ac.kr (J.P.); 2 Department of Food Science and Technology, Chung-Ang University, 4726, Seodong-daero, Anseong-si 17546, Korea; jihlee@cau.ac.kr; 3 Department of Food Science, Sun Moon University, Asan, Chungchengnam-do 31460, Korea; suhhj@sunmoon.ac.kr; 4 School of Food Science, University of Idaho, 875 Perimeter Drive MS 2312, Moscow, ID 83844–2312, USA; hlee@uidaho.edu

**Keywords:** calcium phosphate dibasic, calcium phosphate tribasic, IC analysis, E341(ii), E341(iii), method validation

## Abstract

An analytical method to measure solubilized orthophosphate ions (HPO_4_^2−^ and PO_4_^3−^ ) from the water-insoluble food additives calcium phosphate dibasic (DCP) and calcium phosphate tribasic (TCP) in processed foods was optimized by comparing ion chromatography (IC) coupled with DS6 conductivity detector (Cond.) and high-performance liquid chromatography (HPLC) with Evaporative light scattering detector (ELSD) methods. The ion-pairing HPLC method could analyze calcium and phosphate ions successively. However, this method exhibited low reproducibility after approximately 48 hours of measurements. The IC method was established as an effective method of measuring orthophosphate ions with high reproducibility using distilled water and KOH solution as the mobile phase with a Dionex column. Matrix-based limit of detections (LOD) and limit of quantifications (LOQ) for snacks and cereals were estimated in the range of 0.01–0.91 µg/mL and 0.21–2.74 µg/mL, respectively. In inter-day and intra-day tests, the calculated precision (%RSD) and accuracy (recovery %) ranged from 0.5% to 6.6% and 82% to 117%, respectively, in both food samples. The levels of DCP or TCP could be analyzed in various positive food samples, and the developed IC method demonstrated good applicability in the analysis of DCP and TCP in collected processed foods.

## 1. Introduction

**Figure 1:**
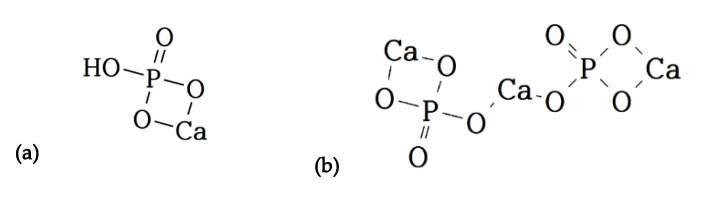
The structural formula of (**a**) calcium phosphate dibasic (DCP) and (**b**) calcium phosphate tribasic (TCP).

Calcium phosphate dibasic (calcium hydrogen phosphate or dicalcium phosphate (DCP)) and calcium phosphate tribasic (tricalcium phosphate (TCP)) are the calcium salt forms of phosphate, which are mainly used as acidity regulators to change or maintain pH in various foods with the EU number of E341(ii) and E341(iii), respectively. The structural formulas of DCP and TCP are shown in Figure 1.

DCP and TCP are used in foods as food additives, such as flour treatment agents and yeast foods, for the purpose of supplying calcium or increasing shelf-life [1,2]. Especially when making bread, DCP and TCP are generally used as a flow conditioner or as a calcium source to enhance nutrition, and they can also be used in feed, dental products and cement [1]. In this study, a survey of the products listed in Korean domestic markets confirmed that they are mainly used for snacks and cereal products. They are colorless and tasteless white crystals, which have molecular formulas of CaHPO_4_·2H_2_O and Ca_3_(PO_4_)_2_ with molecular weights of 136.06–172.09 g/mol and 310.18 g/mol, respectively. These phosphate food additives are insoluble in water and ethanol but soluble in dilute hydrochloric acid and nitric acid, resulting in the formation of orthophosphate anions (HPO_4_^2−^ and PO_4_^3−^) [2]. When left in water for an extended time, DCP is converted to the dihydrate form [3]. The anhydrous form of DCP is more physically stable than the dihydrate form, and it is not well hydrated even when dispersed in water for over 7 months at 4–50 °C [3].

The DCP in foods is slowly absorbed by the small intestine [4], and it can irritate the skin and eyes [5]. The LD_50_ value of TCP for acute oral toxicity is estimated to be greater than 2000 mg/kg body weight in female Wistar rats [6]. An overdose of TCP over the acceptable daily intake (ADI) has been reported to cause maternal toxicity in pregnant Wistar rats, including in the liver, kidneys, and brain [7]. Based on previous studies, the ‘maximum tolerable daily intake’ (MTDI) of both additives was set as 70 mg/kg body weight per day by the JECFA in 1982. The JECFA defined the MTDI rather than the ADI because phosphorus is an essential nutrient and is naturally present in foods, which is consistent with the recommendations of the Scientific Committee for Food (SCF) [8]. Due to the low toxicity of DCP and TCP, they are designated as GRAS substances by the FDA in 1997. Their GMP levels, a reference to the maximum level necessary to technological purposes under conditions of GMP, have also been established in Canada and Australia [9,10]. They are allowed as food additives with very high levels of calcium or phosphorus by CODEX, the EU, and Japan. In Korea, they can also be added to all foods up to 1% as calcium, except for health supplements and special purpose foods [11].

Although DCP and TCP were designated as food additives in many countries, limited validated methods including TLC or spectrophotometry have been developed for measuring the levels of DCP and TCP in foods [6]. This is mainly because of difficulties in distinguishing DCP and TCP with natural phosphates and other types of food additives including phosphorus and because of the complexity of food matrices. Although there are modern analytical methods that can separate and quantify other phosphates using high-performance liquid chromatography (HPLC), ion chromatography (IC), capillary zone electrophoresis (CZE), and nuclear magnetic resonance spectroscopy, natural phosphates in foods cannot be easily distinguished from additives [6]. The market for calcium phosphates is growing, and DCP and TCP are used to fortify calcium and prevent caking or clotting effects [12]. This may lead to an overdose of unnecessary phosphorus in individuals who already consume sufficient levels of phosphorus, eventually resulting in toxicity. Thus far, there have been no reports of quantitative analytical methods for DCP and TCP in processed foods using HPLC or IC. The study to use ion-pairing HPLC with an ELSD as the detector to evaluate phosphate anions (PO_4_^3−^) from phosphoric acid in the shell of *Plastrum testudinis*, a well-known Chinese medicine, was published as a Short Communication in the Journal of Pharmaceutical and Biomedical Analysis [13]. Therefore, the use of calcium phosphate should be regulated, and proper analytical methods should be developed for the estimation of DCP and TCP in processed foods. In this study, modern analytical methods including HPLC-ELSD and IC-DS6 Cond. were compared and optimized to evaluate the levels of DCP and TCP in processed foods, which included method validation and pretreatment methods to distinguish DCP and TCP from other phosphate food additives or natural phosphorus components in processed foods.

## 2. Materials and Methods 

### 2.1. Chemicals and Reagents

Hexanes, HPLC grade water, and acetonitrile (ACN) were purchased from Honeywell Burdick and Jackson (Ulsan, Korea), and hydrochloric acid and calcium chloride anhydrous were obtained from Duksan Pure Chemical (Ansan, Korea). Trifluoroacetic acid (TFA), ammonium acetate, and ammonium formate from Fisher Chemical (Loughborough, UK) were used for HPLC analysis. In addition, sodium hydroxide solution (50% *w*/*w*) and Dionex EGC III KOH for IC analysis were products from Fisher Chemical (USA) and Thermo Scientific (USA), respectively. Heptafluorobutyric acid (HFBA) was purchased from ACROS (Shanghai, China). All reagents for IC analysis were prepared with 18 MΩ resistance water obtained from an ELGA PURELAB Ultra Bioscience water purification system (High Wycombe, UK).

DCP (St. Louis, MO, USA), TCP (St. Louis, MO, USA), phosphate standard solution for IC analysis (Buchs, Switzerland), sodium pyrophosphate decahydrate (Tokyo, Japan), and sodium triphosphate pentabasic (Munich, Germany) were purchased from Sigma-Aldrich. The CAS numbers of the dehydrated form and dihydrate form of DCP are 7757-93-9 and 7789-77-7, respectively, and TCP has a CAS number of 7758-87-4. Sodium phosphate dibasic anhydrous and calcium phosphate monobasic were the products of Samchun Pure Chemical (Pyeongtaek, Korea).

### 2.2. Sample Pretreatment

Several pretreatment conditions were tested by comparing the type of solubilizing solvent and filter paper with an additional drying step. Distilled water (10 mL) was added to the ground food sample (1 g) in a tube. After vortexing for 1 min, the sample was filtered through Whatman filter paper No. 4 (GE Healthcare UK Limited, Amersham, UK)with a vacuum pressure for washing and removing soluble phosphate additives or natural soluble phosphates in food. After three times of washing and filtration, the sample containing water-insoluble DCP or TCP on the filter paper was dried for at least 30 min in a dry oven at 100 °C. Dried DCP and TCP on the filter paper were solubilized in 20 mL of 0.5 M hydrochloric acid with vortexing for 1 min. Hexane (5 mL) was added to remove the lipids in processed foods. After vortexing for another 1 min, the tube containing the sample was centrifuged at 10,000 rpm for 10 min. After filtration of the diluted sample (10× with distilled water) with a syringe membrane filter (pore size 0.45 µm), the filtrate was injected into the HPLC or IC system.

### 2.3. HPLC Analysis

Three modified ion-pairing HPLC methods with an evaporative light scattering detector (ELSD) based on a previous report [13] were compared using Agilent Technologies HPLC 1200 Series (Richardson, Texas, USA) equipped with degasser, binary pump, autosampler, thermostatted column compartment, and a detector of Agilent 1260. Agilent Eclipse XDB-C18 (4.6 × 250 mm, 5 µm; USA) and YMC Triart-C8 (4.6 × 250 mm, 5 µm; Komatsu, Japan) were tested with different solvent ratios. Both isocratic elution conditions which were mobile phases of ACN: 0.7% TFA with 5 mM HFBA in water = 2:98 or ACN: 0.7% TFA with 5 mM HFBA in water = 9:91 were applied with a flow rate of 0.5 mL at 25 °C. The injection volume was 10 µL.

### 2.4. IC Analysis

IC analysis was carried out with ICS 2100 (Thermo Scientific), which consisted of the DS6 Heated Conductivity Cell, autosampler (AS50; Dionex), and gradient pump (GP50; Dionex). The Dionex ASRS 300 (4 mm) suppressor was operated in the auto suppression external water mode. An IonPac AS16 (analytical, 4 × 250 mm) column with a guard column (4 × 50 mm) was attached to the IC system, and sample analysis was performed at 30 °C (IC conditions 1) or 35 °C (IC conditions 2 and 3). The mobile phase was adjusted using EGC III KOH, and the flow rate was set at 1.0 mL/min with a gradient described in Table 1. AutoSuppression Recycle mode was applied in IC condition 1 and 2, and AutoSuppression external water mode was set in IC condition 3. Chemical suppression mode did not apply in this study. Three different chromatographic systems were tested, as shown in Table 1.

### 2.5. Method Validation

Validation was performed to verify the tested HPLC and IC methods with pretreatment methods by evaluating the linearity, limit of detection (LOD), the limit of quantitation (LOQ), precision, accuracy, and recovery. Matrix-based standard curves of DCP and TCP were prepared at 250, 500, 1000, 1500, and 2500 µg/mL and at 6.25, 12.5, 25, 50, and 75 µg/mL for HPLC and IC analysis, respectively. The correlation coefficients in the calibration curve were estimated to examine the linearity, and LOD and LOQ at three low concentrations (6.25, 12.5, and 25 µg/mL) were calculated according to the guidelines of the ICH [14] using the slope and standard deviation of the analytical response (LOD = 3.3 σ/S, LOQ = 10 σ/S, σ = standard deviation based on the results of the intermediate concentration measured seven times, S = slope obtained from the calibration curve to calculate the LOD and LOQ). The precision and accuracy were estimated for the IC method from the data of inter-day and intra-day tests. After the addition of three concentrations (2000, 6000, and 12,000 µg/mL) of DCP and TCP to blank samples, the recovery was observed once a day for three days (inter-day test) and five times in a day (intra-day test) by analyzing the recovery of DCP and TCP in the pre-spiked samples. Precision was expressed as the % RSD (percentage of relative standard deviation), and accuracy was estimated as the recovery percentage (%). The accuracy (recovery) was calculated by averaging the measured values for each concentration.

Precision (% RSD) = (standard deviation/mean) × 100(1)

Accuracy (Recovery %) = (mean of determined value/theoretical value) × 100.(2)

## 3. Results and Discussion

### 3.1. HPLC Optimization for Measuring DCP and TCP

**Figure 2:**
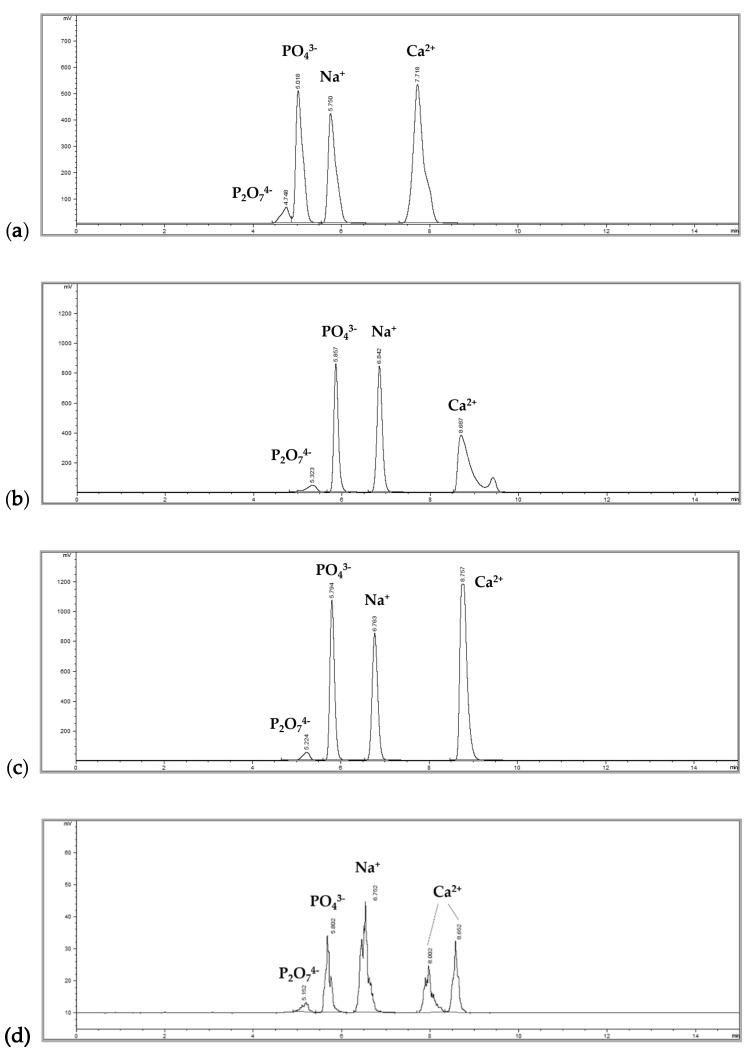
HPLC chromatograms: (**a**) condition 1 with Agilent XDB-C18 at an isocratic mobile phase of acetonitrile (ACN):water (0.7% trifluoroacetic acid (TFA), 5 mM heptafluorobutyric acid (HFBA)) = 2:98; (**b**) condition 2 with YMC Triart-C8 at an isocratic mobile phase of ACN:water (0.7% TFA, 5 mM HFBA) = 2:98; (**c**) condition 3 with YMC Triart-C8 at an isocratic mobile phase of ACN:water (0.7% TFA, 5 mM HFBA) = 9:91; (**d**) after approximately 48 hours of repeated measurements with condition 3.

The authors reported two peaks of phosphate (PO_4_^3−^) and calcium in the HPLC chromatogram with a LOD and LOQ of 7.4 µg/mL and 17.5 µg/mL, respectively, in solvent-based standard curves. However, there was no information on other phosphorus ions such as pyrophosphate. Here, three different HPLC conditions were compared to optimize the method for the analysis of DCP and TCP in foods, and the results are presented in Figure 2. A peak of orthophosphate from DCP, TCP, and phosphoric acid could be separated from the peak of pyrophosphate (P_2_O_7_^4−^) at 1,000 µg/mL under all three HPLC conditions, in which pyrophosphate was eluted as the first peak followed by the peak of orthophosphate, sodium, and calcium. Orthophosphate anions (HPO_4_^2−^ and PO_4_^3−^) from DCP, TCP, and phosphoric acid could not be separated under any HPLC conditions, which showed the same retention time as one peak in the chromatogram. The major differences between HPLC conditions 1 (Figure 2a) and 2 (Figure 2b) and between HPLC conditions 2 and 3 (Figure 2c) were the separation of the peak of pyrophosphate from the peak of orthophosphate and the simultaneous analysis of calcium as one sharp peak.

HPLC condition 3 was selected as the best condition, and the analytical parameters were evaluated further. The calibration curves demonstrated a quadratic equation due to the ELSD in HPLC analysis (data are not shown), and correlation coefficients for DCP and TCP were 0.9998 and 0.9991, respectively, in this polynomial equation. This type of polynomial relationship is often observed with the use of an ELSD as previously reported [15,16]. Interestingly, the calibration curves of DCP and TCP overlapped in one calibration curve (*r*^2^ = 0.9994) when the peak area was plotted according to the phosphate content itself. The LOD and LOQ were 9.09 µg/mL and 9.37 µg/mL, respectively, for DCP in this overlapped calibration curve.

Based on the results, ion-pairing HPLC condition 3 could be used for the analysis of DCP or TCP in the orthophosphate form as an improvement of the previous method [13]. However, this method could not be easily applied to the analysis of DCP and TCP in processed foods because the column used in the analysis had short-term chromatographic reproducibility due to coating with the ion-pairing agent (HFBA). In our study, the peak patterns in the chromatogram were different after approximately 48 hours of running time as shown in Figure 2d, in which the peak area was greatly reduced, and split peaks were observed. This change in the peak pattern was repeatedly observed even after using a new column. Furthermore, the low reproducibility may be attributed to solvents with a very low pH in the column and the difficulty in equilibrating ion-pairing agents [17]. In addition to ion-pairing HPLC, ion-exchange HPLC can also separate anions or cations [18,19]. However, DCP and TCP could not be analyzed easily under any of the tested conditions for ion-exchange HPLC in our study.

### 3.2. IC Optimization for Measuring DCP and TCP

DCP and TCP are insoluble in water, and they can be solubilized in a diluted solution of hydrochloric acid [2]. Thus far, no scientific trials have used IC to measure orthophosphates (HPO_4_^2−^ and PO_4_^3−^) from both water-insoluble DCP and TCP. Previously, IC was used in the analysis of the salts of polyphosphate anions including orthophosphate (PO_4_^3−^), pyrophosphate (P_2_O_7_^4−^), and triphosphate (P_3_O_10_^5−^), which are mainly used as additives in seafood or marine products to increase quality and shelf life [20,21]. However, previous methods were only limited to the measurement of soluble polyphosphate additives in sea products, and the process could not be easily adapted to the analysis of water-insoluble DCP and TCP in processed foods. In order to develop a proper IC method to analyze DCP and TCP in processed foods in the form of solubilized orthophosphate anions, the following two major problems should be addressed: reducing the negative effect of a high concentration of chloride ions from hydrochloric acid on IC analysis and removing other phosphate anions from soluble food additives and natural phosphorus components in foods.

**Figure 3:**
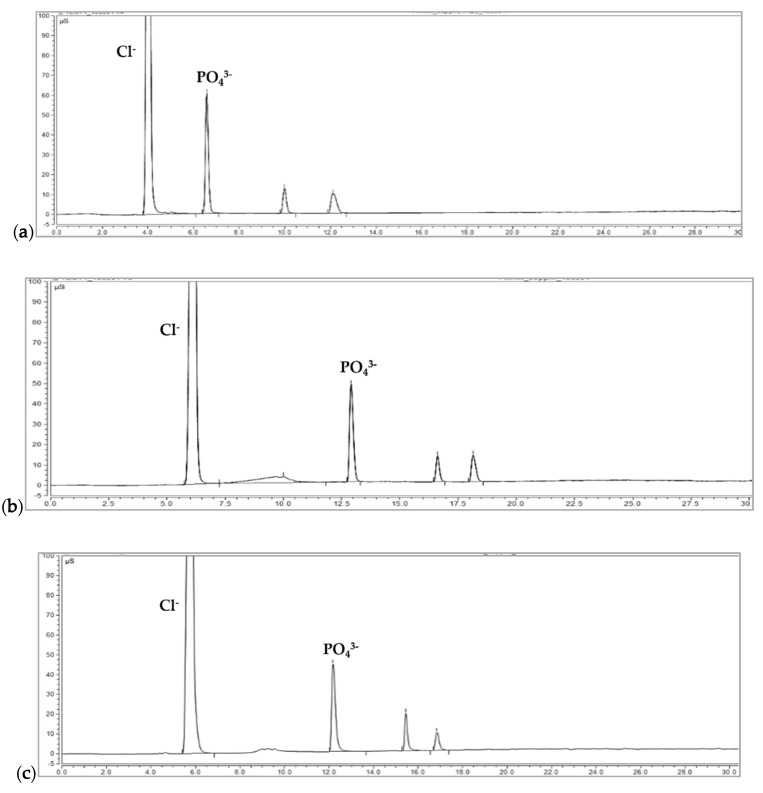
IC chromatograms of standard mixed solutions: (**a**) condition 1 with ASRS 300 as a suppressor at 30 °C; (**b**) condition 2 with ASRS 500 as a suppressor at 35 °C; (**c**) condition 3 with ASRS 300 as a suppressor at 35 °C in the auto suppression external water mode. IonPac AS-16, an analytical IC column (4 × 250 mm), was used for all conditions with a guide column (4 × 50 mm).

After DCP and TCP were solubilized in hydrochloric acid, three different analytical IC methods were examined based on previous studies [20,21] as shown in Figure 3.

An IC method based on a Ministry of Food and Drug Safety (MFDS) report (IC condition 1) [21], which used the AS16 (4 × 250 mm) instead of AS11 (4 × 250 mm) column, showed a very large chloride ion peak in the chromatogram, followed by the peaks of PO_4_^3−^, P_2_O_7_^4−^, and P_3_O_10_^5−^ as shown in Figure 3a. As expected, the peak of the chloride ion was too large, leading to the tailing of the peak, and all polyphosphate anion peaks were detected within 10 min. Orthophosphate anions such as HPO_4_^2−^ and PO_4_^3−^ from DCP, TCP, and phosphoric acid could not be separated from each other, and they were detected as one peak in ion-pairing HPLC analysis (Figure 2). The blockage of flow in the suppressor or cells in the detector was irregularly observed. This might be caused by the formation of insoluble salts of phosphate anions due to the re-binding of solubilized HPO_4_^2−^ and PO_4_^3−^ with calcium ions under alkaline conditions with 90 mM KOH. Ion accumulation can be induced by solvent oxidation in IC analysis [22]. Figure 3b shows the result obtained using the previously reported method [20] with IC condition 2, in which NaOH unlike previously reported conditions [20] was substituted with KOH generated by a KOH eluent generator (EGC III KOH). The peak of orthophosphate anions (HPO_4_^2−^ and PO_4_^3−^ peaks) was detected at 12 min, followed by the peaks of P_2_O_7_^4−^ and P_3_O_10_^5−^. Similar to IC condition 1, a large peak of chloride ions from hydrochloric acid was observed at the beginning of the elution, followed by the orthophosphate anion peak with a relatively long distance from the first chloride ion peak as shown in Figure 2b. This method also could not be applied further to the analysis of orthophosphate anions (HPO_4_^2−^ and PO_4_^3−^) from DCP and TCP even though it demonstrated excellent peak shape and peak resolution. Similar to IC condition 1, the measurements could not be continued due to the irregular blockage of flow in the suppressor or cells in the detector. To overcome the problems described for IC condition 1 and 2, an additional process was included in the IC analysis system (IC condition 3). The blockage of flow in the suppressor or cells in the detector could be reduced by the auto suppression external water mode, in which the membrane in the suppressor was washed out continuously by external water. As shown in Figure 3c, the excellent separation of polyphosphate ions between peaks was observed. The auto suppression external water model was used to reduce accumulated ions in the detector or suppressor that can occur irregularly. This mode is recommended for analysis of high matrix ion concentrations to reduce noise by removing more counter ions from the eluent and the occurrence of ion deposits resulting from solvent oxidation [22,23]. IC condition 3 was finally selected as the optimal condition in this study for measuring orthophosphate anions from DCP and TCP.

### 3.3. Pretreatment of DCP and TCP in Processed Food Samples for IC Analysis

Most phosphate-containing food additives approved in Korea are water-soluble salts, and DCP and TCP are the only approved water-insoluble food additives containing phosphate anions except ferric phosphate, which is rarely used in processed foods [24]. Water-insoluble characteristics are crucial because they can be beneficial in the pretreatment procedure for IC analysis, which removes most soluble food additives or food components containing phosphate or phosphorus in processed foods. This strategy is comparable to previously reported pretreatment methods that analyze soluble polyphosphate additives from seafood or marine products [20,21]. Water-soluble polyphosphate ions in seafood were extracted with water after the inactivation of phosphatase by heating for 10 min at 100 °C or microwaving for 40 s at 750 W, and the extracted polyphosphate ions were analyzed by IC [20,21]. These pretreatment methods are an extraction procedure for only water-soluble additives.

DCP and TCP in processed foods are water-insoluble phosphate salts, and they can be separated from soluble phosphate additives and natural soluble phosphorus in foods before the solubilization of DCP and TCP with hydrochloric acid. Eventually, the phosphate anions from soluble food additives and natural phosphates in processed foods are easily removed in the first step after the homogenization of processed food samples containing DCP or TCP by washing several times with water using Whatman filter paper. Various types of filter papers including Whatman No. 3, 4, and 42 were tested to recover water-insoluble DCP or TCP during washing, and it was determined that Whatman filter paper No. 4 or a filter with a smaller pore size is sufficient for recovering DCP or TCP in food samples. After the separation of DCP and TCP by filtration, the samples on the filter were fully dried in a dry oven for 30 min at 100 °C to avoid the dilution of added hydrochloric acid for solubilizing DCP and TCP in the next step and to calculate the dilution fold accurately. This drying step is critical for the good recovery of DCP and TCP from processed food samples. After this pretreatment procedure, spiked DCP in blank food samples could be recovered successfully with a recovery ratio of 85.3%.

### 3.4. Method Validation for IC-Cond

Method validation for a developed analytical method is a systematic process of verifying the acceptability of a method based on several parameters, such as the linearity, LOD, and LOQ, where it is determined whether the analytical method is well suited for experimental purposes [25,26]. Although not all variables that may cause problems can be removed through method validation, the method can be controlled during reproduction [27]. Therefore, the previous IC analytical method for polyphosphates was not suitable for application due to the lack of validation data [20]. In this study, the parameters linearity, LOD, LOQ, precision, and accuracy were calculated for method validation in two types of the food matrix, i.e., snacks and cereals, which typically contain DCP or TCP as an acid regulator.

#### 3.4.1. Linearity

**Figure 4:**
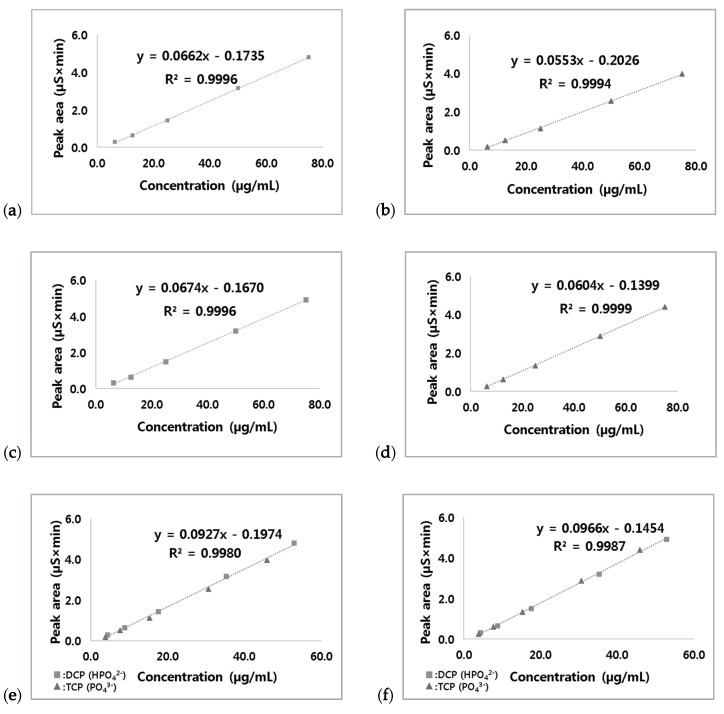
Matrix-based calibration curves: (**a**) DCP in snacks; (**b**) TCP in snacks; (**c**) DCP in cereals; (**d**) TCP in cereals; (**e**) DCP (HPO_4_^2−^) and TCP (PO_4_^3−^) in snacks; (**f**) DCP (HPO_4_^2−^) and TCP (PO_4_^3−^) in cereals.

The concentration results of the analytical methods should be proportional and linear [28]. Matrix-based calibration curves of DCP and TCP in snacks and cereals were established for IC analysis at various concentrations as shown in Figure 4. All standard curves showed good linearity with a correlation coefficient (*r*^2^) of more than 0.997. When the peak area in IC analysis was plotted according to the phosphate content only without calcium (29.5% and 38.8% for DCP and TCP, respectively), the calibration curves of DCP and TCP could be merged in one graph, which exhibit a *r*^2^ of 0.9980 and 0.9987 for the snack (Figure 4a,b) and cereal samples (Figure 4c,d), respectively, as shown in Figure 4e,f. Similar to HPLC analysis, orthophosphate ions (HPO_4_^2−^ and PO_4_^3−^) could not be separated even in IC analysis. Therefore, this merged standard curve can be used for the analysis of phosphate anions from the unknown water-insoluble salts of orthophosphates (e.g., DCP or TCP in unlabeled food samples or positive food samples labeled with a mix of acid regulators including DCP and TCP).

#### 3.4.2. LOD and LOQ

According to the FDA [26], the LOD is the lowest concentration that can be determined as statistically different from a blank at a specified level of confidence, and the LOQ is the lowest amount or concentration of an analyte that can be quantitatively determined with an acceptable level of uncertainty. The matrix-based LODs of DCP were estimated as 0.79 µg/mL and 0.91 µg/mL for snack and cereal samples, respectively. The LODs for TCP were 0.01 µg/mL and 0.32 µg/mL for the same samples. The LOQs of DCP for the same matrices were 0.21 µg/mL and 0.97 µg/mL, respectively. The LOQs of TCP were 2.40 µg/mL and 2.74 µg/mL in snack and cereal samples, respectively. The LODs and LOQs for DCP and TCP showed different values depending on the type of the food matrix. The type of food matrix is an important factor for obtaining excellent results in IC analysis. Therefore, the proper pretreatment method should be prepared as described in Section 3.2 (IC optimization for measuring DCP and TCP).

#### 3.4.3. Precision and Accuracy

Precision indicates the degree of difference in repeated measurements [28], and it is acceptable below 20% [29]. Accuracy is a measure of the degree of conformity of a value generated by a specific procedure to the assumed or accepted true value [26]. The precision and accuracy of IC analysis for DCP and TCP were determined in snack and cereal samples as shown in Table 2. In inter-day and intra-day tests, the precision (% RSD) was in the range of 0.5%–6.6%, and the accuracy was between 82.0% and 116.7%. Thus far, no IC studies have described all essential parameters in validation tests, as observed in a previous study using an IC method to measure polyphosphate ions [20,21].

### 3.5. Sample Analysis

**Figure 5:**
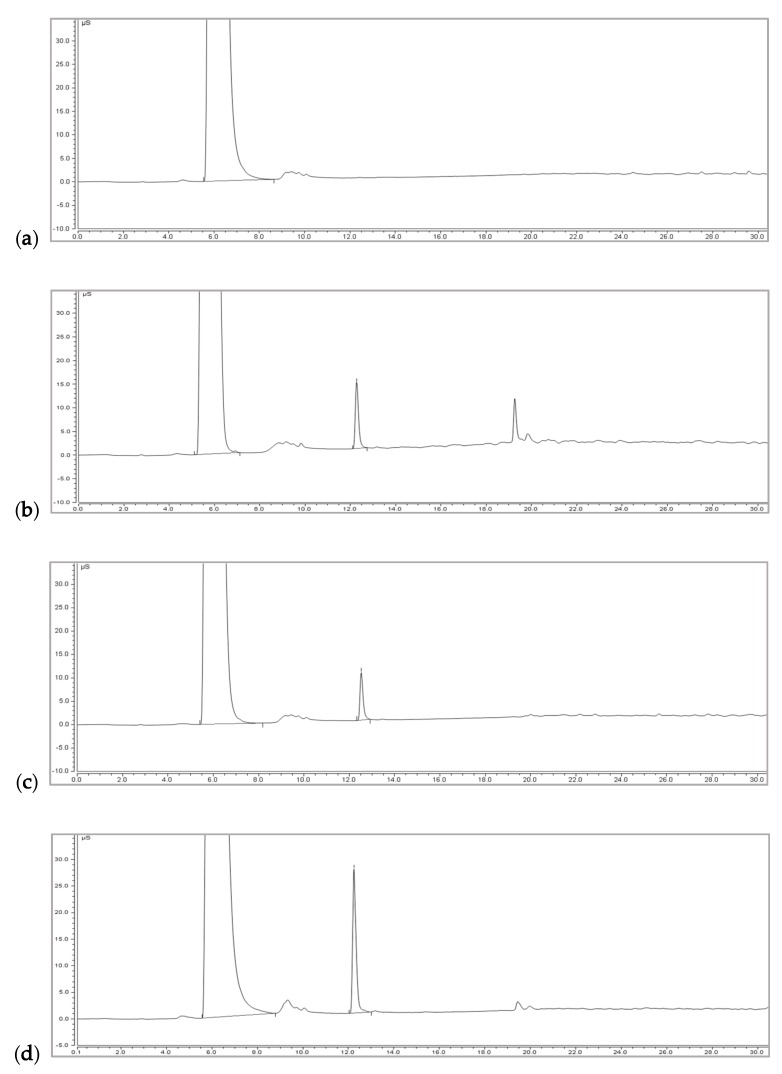
IC analysis of collected processed food samples: (**a**) negative sample; (**b**) positive sample labeled with DCP; (**c**) positive sample labeled with TCP; (**d**) positive sample labeled with a mix of DCP with TCP.

A total of 67 processed domestic and imported snack including biscuit, cookie, and cracker and cereal samples such as pasta, bread, and cereal products labeled with DCP (23), TCP (39), and DCP mixed with TCP (5)) were collected in Korea, and the levels of DCP and TCP were evaluated using the corresponding calibration curves in Figure 4 based on the type of food samples. The results are shown in Figure 5. Positive samples with labels showing the addition of DCP had a DCP level in the range of 7611–14,735 µg/mL for snacks and 1260–17,108 µg/mL for cereals as demonstrated by the chromatograms of a DCP-positive snack sample (Figure 5b). TCP levels in TCP-labeled positive snacks and cereals were detected in the range of 1040–5850 µg/mL and 680–2315 µg/mL, respectively (Figure 5c). If the labels of positive samples showed a mix of DCP and TCP, a single linear equation (Figure 4e) for orthophosphate was used for the calculation, and the results were converted into the level DCP or TCP. For example, a food sample with a mix of DCP and TCP exhibited one peak in the IC chromatogram as shown in Figure 5d, in which orthophosphate ions (HPO_4_^2−^ and PO_4_^3−^) from DCP and TCP could not be separated. If the levels of orthophosphate ions were converted to DCP and TCP levels, the estimated values were in the range of 16,061–17,358 µg/mL and 18,306–19,784 µg/mL, respectively, using the calibration curves in Figure 4e,f. In sample analysis, a high concentration of hydrochloric acid was used for the solubilization of DCP and TCP. The extract should be diluted at least 200-fold during sample pretreatment for IC analysis, and the dilution led to an increase in LOQs up to approximately 600 µg/mL during the calculation. Therefore, the small orthophosphate peak in negative samples below 1000 µg/mL (presumed to be naturally existing phosphate in foods) could be negligible. The high levels of DCP and TCP up to 20,000 µg/mL in positive processed food samples could be clearly differentiated from the values calculated for the negative samples.

## 4. Conclusions

The ion-pairing HPLC and IC methods were compared and optimized to develop analytical methods to quantify DCP and TCP in the form of orthophosphate ions in processed foods. IC analysis showed reliable and reproducible results, and an optimized condition was finally selected with proper pretreatment conditions to separate DCP and TCP from other phosphorus compounds in processed foods. This novel optimized method was verified with reliable validation parameters. The IC method used in this study with pretreatment conditions for DCP and TCP can be applied to the analysis of these food additives in processed foods.

## Figures and Tables

**Table 1:** Technical details of the three ion chromatography (IC) systems.

Parameter	IC condition 1		IC condition 2		IC condition 3	
Column	Thermo Scientific IonPac AS-16, analytical (4 × 250 mm) Thermo Scientific IonPac AG-16, guard (4 × 50 mm)					
Column temp.	30 °C		35 °C		35 °C	
Mobile phase	EGC ^a^ III KOH					
	Time (min)	KOH (mM)	Time (min)	KOH (mM)	Time (min)	KOH (mM)
	0 2 20 24 31 33 35	20 40 80 100 100 20 20	0 5 22 32 35 42	2.6 20 100 100 2.6 2.6	0 5 20 30 33 38	3 20 90 90 3 3
Flow rate	1 mL/min		1 mL/min		1 mL/min	
Suppressor	ASRS ^b^ 300 (Dionex, 4 mm)		ASRS ^b^ 500 (Dionex, 4 mm)		ASRS ^b^ 300 (Dionex, 4 mm), Auto-suppression external water mode	
Injection vol.	25 µL		25 µL		25 µL	
Run time	35 min		42 min		38 min	

^a^ EGC: Eluent Generator Cartridges; ^b^ ASRS: Anion Self-Regenerating Suppressor.

**Table 2:** Precision and accuracy determined from inter-day and intra-day tests of the snack and cereal matrix.

		Concentration (µg/mL)		2000		6000		12,000	
Method				DCP	TCP	DCP	TCP	DCP	TCP
Snack	Inter-day		Precision	3.9	1.8	1.5	1.3	2.3	4.2
			Accuracy	103.7	100.2	92.6	95.2	82.0	88.8
	Intra-day		Precision	3.8	4.8	0.8	1.4	1.9	2.7
			Accuracy	95.9	101.0	84.4	97.6	86.0	97.2
Cereal	Inter-day		Precision	1.3	1.3	1.6	0.7	6.6	0.7
			Accuracy	116.1	114.2	101.1	107.3	88.5	105.4
	Intra-day		Precision	1.1	0.5	0.6	2.7	1.6	1.4
			Accuracy	116.7	113.9	102.2	100.3	98.4	99.2

Inter-day test (once a day for three days) and intra-day test (five times in a day) were performed using pre-spiked samples at three concentrations. Precision (% RSD) = (standard deviation/mean) × 100; Accuracy (Recovery %) = (mean of determined value/theoretical value) × 100. DCP: calcium phosphate dibasic; TCP: calcium phosphate tribasic.
